# Challenges to replace ACT as first-line drug

**DOI:** 10.1186/s12936-017-1942-5

**Published:** 2017-07-24

**Authors:** Aung Pyae Phyo, Lorenz von Seidlein

**Affiliations:** 1 Shoklo Malaria Research Unit, Mahidol-Oxford Tropical Medicine Research Unit, Faculty of Tropical Medicine, Mahidol University, Mae Sot, Thailand; 2 Mahidol-Oxford Tropical Medicine Research Unit, Faculty of Tropical Medicine, Mahidol University, Bangkok, Thailand

## Abstract

The spread of artemisinin and partner drug resistance through Asia requires changes in first-line therapy. The traditional modus has been the replacement of one first-line anti-malarial regimen with another. The number of anti-malarial drug candidates currently in development may have given false confidence in the expectation that resistance to artemisinin-based combination therapy (ACT) can be solved with a switch to the next anti-malarial drug regimen. A number of promising anti-malarial drug regimens did not succeed in becoming first-line drugs due to safety concerns or rapid development of resistance. Currently promising candidates for inclusion in first-line regimens, such as KAE 609, KAF 156, OZ 439, and OZ 277, have already triggered safety concerns or fears that point mutations could render the drugs inefficacious. An additional challenge for a new first-line drug is finding an appropriate partner drug. There is hope that none of the above-mentioned concerns will be substantiated in larger, upcoming trials. Meanwhile, combining already licensed anti-malarials may be a promising stop-gap measure. Practitioners in Vietnam have empirically started to add mefloquine to the current dihydroartemisinin-piperaquine. Practitioners in Africa could do worse than empirically combine already licensed co-artemether and amodiaquine when treatment with ACT no longer clears *Plasmodium falciparum*. Both combinations are currently undergoing trials.

## Background

The spread of artemisinin and partner drug resistance through Asia requires changes in first-line therapy. In Binh Phuoc Province, Vietnam, the proportion of malaria patients with detectable falciparum parasitaemia after three days of treatment with the current first-line treatment, dihydroartemisinin piperaquine, has reached 57% and the parasite clearance half-life doubled from 3.75 h in 2011 to 6.60 h in 2015 [1]. The now dominant artemisinin-resistant *Plasmodium falciparum* C580Y lineage in the Greater Mekong Sub-region (GMS) probably arose in western Cambodia and spread through Vietnam, Laos and Thailand, outcompeting other parasites and acquiring piperaquine resistance [2]. West of the GMS, *P. falciparum* strains with two non-synonymous mutations in the *k13* propeller region have been detected in Changlang district, Arunachal Pradesh, India. While treatment outcome has not yet been affected in India, the appearance of phenotypic resistance is probably only a question of time. In the past, chloroquine (CQ) and later sulfadoxine/pyrimethamine (SP)-resistant *P. falciparum* strains spread from India to East Africa and then through the African continent. The recent emergence and spread of multidrug-resistant *P. falciparum* strains, which are no longer cured by artemisinin-based combination therapy (ACT), has resulted in considerable research, with support from large funders (e.g., Global Fund, Bill and Melinda Gates Foundation, and Wellcome Trust) without any notable impact. When CQ was compromised by resistance, the attributable mortality in sub-Saharan Africa in the 1980–1990s was millions [3]. Yet the spread of ACT resistance has not resulted in the declaration of a Public Health Emergency of International Concern (PHEIC). This omission could be explained by an overreliance on novel anti-malarial drug candidates, which could potentially replace ACT as first-line treatment. This paper discusses potential flaws of such candidate drugs.

## Replacement for ACT?

The previous modus of replacing one first-line anti-malarial regimen with another may not be possible now. There is a range of reasons why promising, efficacious anti-malarials in the pipeline did not become first-line treatments. Halofantrine induces consistent dose-related lengthening of the PR and QT intervals, which made it unsuitable for further use in public health [4]. Pyronaridine, in the combination with artemisinin, was considered a highly promising combination therapy until a data monitoring committee found evidence of elevated transaminases in a pivotal trial consistent with low-level toxicity [5]. Further studies suggested that the risk of a hepatotoxicity event was no greater after pyronaridine–artesunate retreatment than after first treatment (0.19 vs 0.54%) yet an aspartate transaminase (AST) increase (3% in first and retreatments) as well as an increased alanine transaminase (ALT; 3% in first and 2% retreatments) with this combination is still higher than in artemether-lumefantrine treatment [6].

The European Medicines Agency has since granted a revised label for the pyronaridine-artesunate combination, removing all restrictions on repeat-dosing, on use only in areas of high resistance and low transmission, and on requirements for liver function monitoring. However the drug’s disappointing efficacy, <90% in Western Cambodia [7], has cast doubt about the usefulness of the pyronaridine–artesunate combination as an universal first-line treatment. Atovaquone was introduced in 1990 and did not become a first-line treatment probably due to its high cost. In addition, atovaquone is particularly susceptible to resistance as single nucleotide polymorphisms can confer high-level resistance. Atovaquone resistance was reported within a year of introduction [8].

The recent publication of promising trials with several new anti-malarial drug classes has given a reassuring impression that the “anti-malarial drug development pipeline has probably never looked healthier” [9] (see Table 1). Based on the flaws that derailed progress of earlier, promising anti-malarial drugs, there are reasons to be sceptical about the promise of the following candidates in the pipeline:

**Table 1:** Critical characteristics of four potential antimalarial drug candidates

	KAE609	KAF156	OZ439	OZ277
Potential partner drug?	Ferroquine	Solid dispersion formulation-lumefantrine	Piperaquine ferroquine	Piperaquine
Drug half-life (h)	21	48–60	46–62	2–4
Efficacy	PCt1/2 0.9 h	PCt1/2 3.5 h	PC1/2 = 4.4 (WT K13) PC1/2 = 5.5 (Mutant)	Median PCt = 24 h same as coartem
			PCR-corrected ACPR on day 42 was under 50% with single dose piperaquine	PCR-corrected ACPR on day 42 was >98% in both groups
Transmission blocking	Reduction in stage 5 gametocyte in vitro and inhibit oocyst development in standard membrane feeding assay	Reduction in stage 5 gametocyte in vitro and inhibit oocyst development in standard membrane feeding assay	7 patients with pre-treatment and 10 patients with post-treatment Pf gametocytes; one had up to day-6	Day 7 and Day 14 carriage higher than coartem [23]
Adverse event	Raised transaminase (3/21 patients)	Raised transaminase (30% of patients)	12/82 patients increased CPK	6.9% (49) got QTcF > 500 ms compared to 1.7% (6) in coartem
			7/82 patients increased transaminases	
			2/82 patients QTcF > 450 ms. 18/82 patients with prolongation of QTcF > 30 ms from baseline	
Resistance	SNP at PfATP4 gene can be selected in vitro after 4-months exposure to incremental increasing conc. of KAE	SNP at PfCARL gene can be selected in vitro after 4-months exposure to incremental increasing conc. of KAF	Slower PCt1/2 in K-13 mutation even though in vitro RSA suggested no difference	Not tested
MIC	0.1 ng/ml		4.1 ng/ml [17]	

Conc., concentration; K13, PF3D7_1343700 kelch propeller domain; MIC, minimal inhibitory concentration; ng, nanogram; ml, millilitre; ms, millisecond; PCR, polymerase chain reaction; PCt, parasite clearance time; Pf, *Plasmodium falciparum*; PHEIC, Public Health Emergency of International Concern; RSA, ring-stage survival assay; SNP, single nucleotide polymorphism; WT, wild type

- KAE 609, Cipargamin™, (a phase-2 compound), is the most advanced candidate targeting ATPase4. It has the shortest parasite clearance time (PCt ½ < 1 h) observed in any anti-malarial drug [10]. A major concern is single nucleotide polymorphisms (SNP) at the PfATP4 gene which can be selected in vitro after 3–4 months exposure to incrementally increasing sub-lethal concentrations of KAE 609 [11]. If this finding is substantiated, the loss of KAE 609, like atovaquone earlier, seems likely. A second concern about KAE 609 is that administration resulted in abnormal liver function in 3 of 21 [12], alkaline phosphatase increase in 10 of 25, ALT increase in 1 of 25 and hyper-bilirubinaemia in 5 of 25 [13] study participants.
- KAF 156, an imidazolopiperazine (a phase-2 compound), has a slower PCt rate than KAE 609 but presents similar concerns [12, 14, 15]. Single nucleotide polymorphisms (SNP) in PfCARL, the likely drug target of KAF 156, can be selected in vitro after 4 months exposure to incrementally increasing concentrations of KAF [16]. In addition, 30% of study participants had elevated transaminases.
- OZ 439, Artefenomel™, (a phase-2 compound), belongs to the ozonides class of drugs, which share peroxide pharmacophore with artemisinins [17, 18]. Like artemisinins, the PCt 1/2 of OZ439 is slower in parasites with *k13* mutation suggesting cross-resistance between ozonides and artemisinins [18]. There are safety concerns, as trial participants receiving the drug candidate had elevated creatine phosphokinase (CPK), transaminases and a QTc interval prolongation [18]. A study of 60 volunteers, given a single dose regimen combining 800 mg OZ439 with 960 or 1440 mg piperaquine, is expected to result in lower peak piperaquine plasma concentrations compared with available 3-day piperaquine-artemisinin combinations and can therefore be expected to cause less QTc prolongation [19].
- OZ 277, Arterolane™, (a phase-4 compound); another synthetic peroxide is the most advanced of the new anti-malarials. Combined with piperaquine, this new drug is already registered and marketed by Ranbaxy in India [20–22]. There is a concern that OZ277 shares cross-resistance with the artemisinin derivatives. High resistance levels against the partner drug piperaquine have already been documented in Cambodia and Vietnam. A further concern is the QTc prolongation observed in 6.9% of study participants [23].

It is critical to find and appropriate partner drug for candidate drugs. Ideally the partner drug should facilitate a single dose treatment as SP did in the last century. None of the potential partner drugs can be given as single dose regimen.

## Conclusions

There is hope that none of the above-mentioned concerns will be substantiated in larger, upcoming trials. What can be done besides hoping? Attempts to regain efficacy of artemisinins by increasing dose were disappointing [24] and since only fixed-dose combinations are licensed, increased partner drug dosing could be associated with increased toxicity and adherence issues. Extending the course of routine 3-day treatment to 5 or 7 days was successful, associated with a cure rate of 98% in western Cambodia, the epicentre of drug resistance [25]. The cost of such compromised regimens will increase, while adherence could decrease. Practitioners in Vietnam have instead empirically started to add mefloquine to the current DHA-piperaquine first-line treatment. There appears to be an inverse susceptibility between piperaquine and mefloquine. A similar inverse susceptibility between co-artemether (artemether/lumefantrine) and amodiaquine could be exploited as stopgap measure in Africa [26]. Mefloquine did not gain wide acceptance in Africa where co-artemether is the most popular ACT and there has been extensive experience in the use of amodiaquine. Practitioners could do worse than empirically combine co-artemether and amodiaquine until a replacement for the current first-line treatment becomes available. The safety and efficacy of triple therapy is under evaluation in a series of trials in Asia and Africa [27].

How long stop-gap measures can hold back and prevent increasing morbidity and mortality attributable to ACT resistance is unknown. Meanwhile, ACT resistance is spreading across international borders. Large populations in sub-Saharan Africa whose immunity to malaria is declining with decreasing malaria incidence will be severely affected by the spread of ACT resistance. There are no tried and tested approaches to stop the spread of ACT resistance, but recognizing that the spread of ACT resistance is a public health emergency and of international concern is a necessary step to coordinate an international response.

## Abbreviations

ACPR: adequate clinical and parasitological response
ACT: artemisinin combination therapy
ALT: alanine transaminase
AST: aspartate transaminase
Conc.: concentration
CPK: creatine phosphokinase
CQ: chloroquine
DHA: dihydroartemisinin
GMS: Greater Mekong Subregion
K13: PF3D7_1343700 kelch propeller domain
MIC: minimal inhibitory concentration
ml: millilitre
ms: millisecond
ng: nanogram
PCR: polymerase chain reaction
PCt: parasite clearance time
Pf: *Plasmodium falciparum*
PHEIC: Public Health Emergency of International Concern
RSA: ring-stage survival assay
SNP: single nucleotide polymorphisms
SP: sulfadoxine/pyrimethamine
WT: wild type
